# Intravitreal Ranibizumab Had Limited Effect on Cystoid Macular Edema Due to Albumin-Bound Paclitaxel: A Case Report and Literature Review

**DOI:** 10.3389/fonc.2021.773540

**Published:** 2021-12-13

**Authors:** Suna Ye, Qiqi Fang, Jinyu Yao, Jianqiang Xing, Shibo Tang, Jacey Hongjie Ma

**Affiliations:** ^1^ AIER Eye Hospital, Jinan University, Guangzhou, China; ^2^ AIER Eye Institute, Changsha, China; ^3^ Retina Department, Hainan AIER Eye Hospital, Haikou, China

**Keywords:** albumin-bound paclitaxel, Nab-paclitaxel, Abraxane, cystoid macular edema, ranibizumab, carbonic anhydrase inhibitor

## Abstract

Angiographically silent cystoid macular edema (CME) is a rare complication from nab-paclitaxel. Here we report a 45-year-old woman with breast cancer who developed CME after several months of treatment with albumin-bound paclitaxel (nab-paclitaxel). Her visual acuity did not improve significantly with the cessation of nab-paclitaxel and intravitreal ranibizumab treatment. Then, brinzolamide eye drops were prescribed. One month later, her vision improved, with the macular edema significantly subsided. Finally, we reviewed other cases of CME induced by nab-paclitaxel that have been reported in the literature and discussed the underlying pathogenesis of nab-paclitaxel-induced CME.

## Introduction

Paclitaxel is a mitogenic inhibiting anti-microtubule agent used as first- or second-line treatment in various cancers, alone or in combination with other drugs. The most common indications of paclitaxel include breast cancer, ovarian cancer, and non-small cell lung cancer (1). Albumin-bound paclitaxel (nab-paclitaxel, Abraxane) is an albumin-stabilized paclitaxel nanoparticle formulation with potent antitumor effects than paclitaxel in metastatic breast cancer that has failed combination chemotherapy or recurred within 6 months of adjuvant chemotherapy (2). Cystoid macular edema (CME) is a rare complication caused by nab-paclitaxel (3, 4). Here we report the first case of nab-paclitaxel-induced CME treated with intravitreal ranibizumab (IVR) and review the literature about nab-paclitaxel-related CME.

## Case Report

A 45-year-old female patient diagnosed with right breast cancer underwent a right lumpectomy in March 2016. The pathological examination revealed grade II non-specific invasive ductal carcinoma. The immunohistochemical staining results were as follows: ER (+), PR (–), and HER2(–). The patient received postoperative treatment with traditional Chinese medicine and tamoxifen. In January 2018, the cancer progressed and metastasized to the pectoralis major muscle, lymph nodes, and scapula. From January 2018 to July 2019, she received a chemotherapy regimen comprised of docetaxel, epirubicin, and cyclophosphamide. In July 2019, she started a T regimen nab-paclitaxel (200 mg weekly), which was lowered to 11 consecutive single doses of 170 mg weekly due to adverse drug reactions. From November 12, 2019 to December 25, 2020, the chemotherapeutic regimen of the patient was changed to nab-paclitaxel (once at 400 mg and then thrice at 340 mg, once every 3 weeks). It was then switched to 360 mg once every 3 weeks for a total of 16 doses. On January 16, 2021, she received the last T regimen of nab-paclitaxel (400 mg, cumulative dose: 9,650 mg).

**Figure 1:**
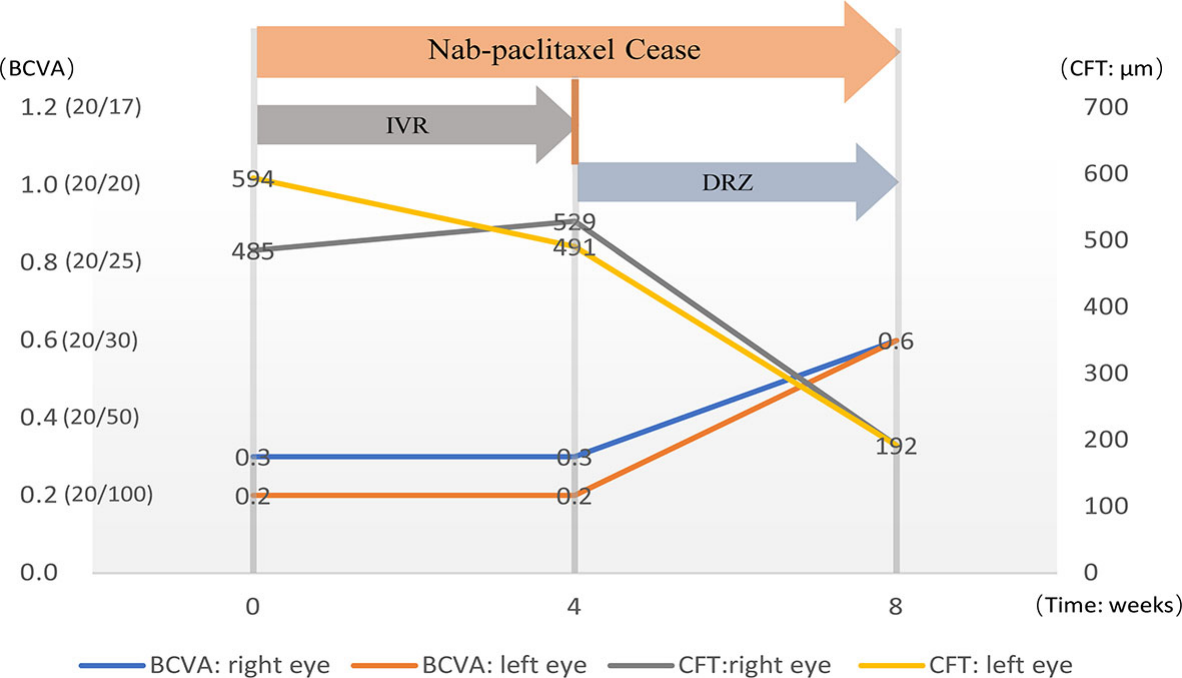
The diagram shows the timeline of treatment and the changes in visual acuity and central retinal thickness.

**Figure 2:**
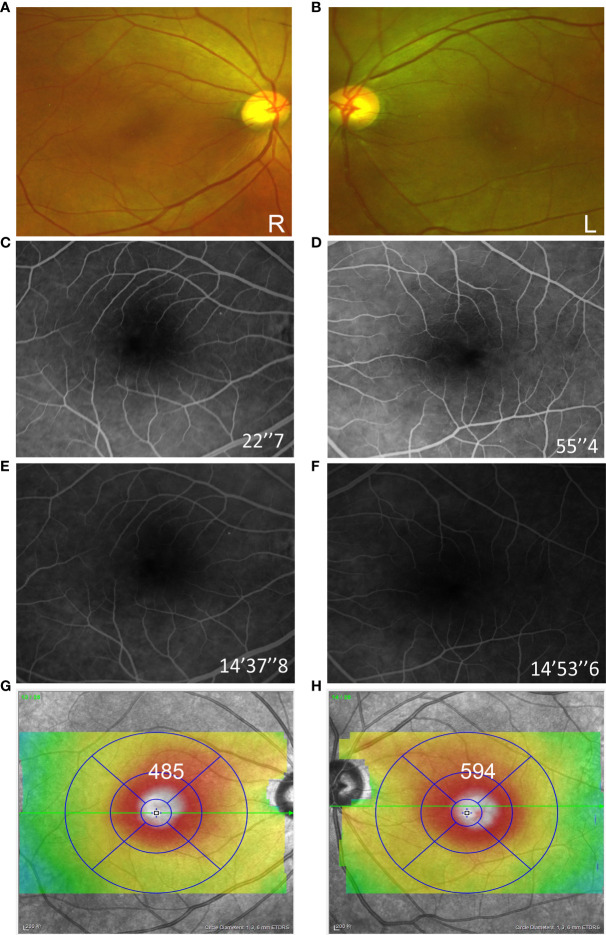
Fundus examination of both eyes at the initial consultation. Ultra-wide field fundus image revealed macular edema in both eyes **(A, B)**. The fluorescein angiograms did not reveal leakage from the parafoveal capillaries **(C–F)**. Optical coherence tomography (OCT) B-scans showed the cystoid edema with a foveal thickness of 485 µm on the right and 596 µm on the left **(G, H)**.

**Figure 3:**
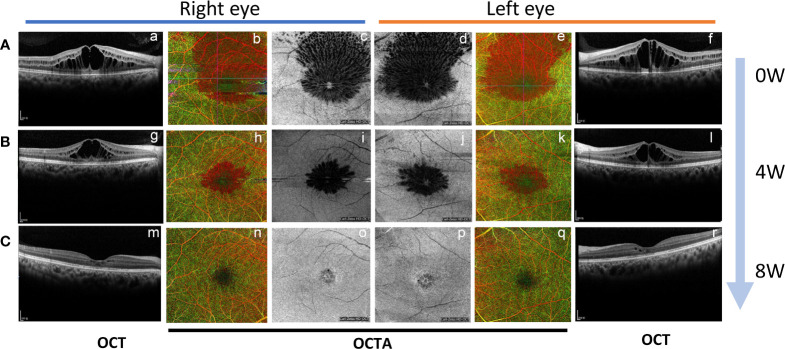
The change of macular edema by B-scan and *en face* optical coherence tomography and OCTA during the time course. **(A)** The OCT B-scan (a, f), OCTA (b, e), and en face OCT (c, d) revealed macular edema in both eyes at initial consultation; **(B)** One month after applying IVR (OCT B-scan g and l, OCTA h and k, en face OCT i and j); **(C)** One month after topical brinzolamide in both eyes. (OCT B-scan m and r, OCTA n and q, en face OCT o and p).

In January 2021, the patient complained of gradually decreasing vision in both eyes over 4 weeks. Then, she received treatment and follow-up by ophthalmologists, and the time course is shown in **Figure 1**. At the initial ophthalmologic examination, the best-corrected visual acuity (BCVA) was 20/60 on the right and 20/100 on the left, and color vision was intact. The anterior segment examination revealed diffuse corneal epithelial defects. The dilated fundus examination revealed macular edema in both eyes (**Figures 2A, B** **)**. The fluorescein angiograms did not reveal leakage from the parafoveal capillaries in early or late stages (**Figures 2C–F**). The optical coherence tomography (OCT) and optical coherence tomography angiography (OCTA) scans on both eyes revealed CME with a thickened central retina (**Figures 2G, H**, **3A**). Neither CME-related systemic nor intraocular disorder, such as diabetes, uveitis, or retinal vein occlusion, was detected after a detailed physical examination and fluorescent angiography. Moreover, she had not taken any other CME-related medications. Then, the diagnosis of taxane-related CME (T-CME) was established.

Nab-paclitaxel was ceased after the CME was revealed, and 1 mg/0.1 ml of ranibizumab (Lucentis, 10mg/ml) was injected intravitreally in both eyes. However, at 1 month after applying IVR, neither the BCVA of the patient nor the central retinal thinness detected by OCT was changed (**Figures 1**, **3A** **-**a, f, **3B**-g, l). However, the area of macular edema was reduced (**Figures 3A**-b–e, **B**-h–k). Since the BCVA was not increased, the patient refused IVR treatment. Then, brinzolamide eye drops (Azopt, 10mg/ml, twice daily) were applied to both eyes. One month later, the BCVA had improved to 20/30 in both eyes, and OCT and OCTA revealed that the macular edema almost subsided in both central macular B-scan (**Figure 3C**-m, r) and *en face* OCT and OCTA (**Figure 3C** **-**n–q)

Written informed consent was obtained from the patient for the publication of any potentially identifiable images or data included in this article.

## Literature Review

We performed a literature search in PubMed and Web of Science before July 30, 2021 for the following terms: “cystoid macular edema” AND (“nab-paclitaxel” or “albumin-bound paclitaxel” or “Abraxane”). A total of 22 nab-paclitaxel-related CME cases were identified in the literature review. The images, treatments, and recovery time of these cases and of our case are presented in **Table 1**.

**Table 1:** Case reports of cystoid macular edema due to albumin-bound paclitaxel.

No.	Author	Sex	Age(years)	Affected eye	Primary tumor	Onset latency (months)	Nab-paclitaxel Case	Other treatments	Treatment time to recovery (months)
1	Risard et al. (5)	Male	58	Bilateral	Melanoma	4	Yes	NO	1.5
2	Haider et al. (6)	Male	73	Bilateral	Lung	42	Yes	NO	2
3	Fenicia et al. (7)	Female	40	Bilateral	Breast	4	–	DEX and DRZ	–
4	Rahman et al. (8)	Female	73	Bilateral	Breast	3	No	IVB^a^	Lost visit
5	Smith et al. (9)	Female	56	Bilateral	Breast	27	Yes	NO	3
6	Murphy et al. (10)	Female	65	Bilateral	Breast	1.5	Yes	NSAID and GC	0.75
7	Murphy et al. (10)	Female	58	Bilateral	Breast	11	Yes	NO	3
8	Rahimy and Sarraf (11)	Female	32	Bilateral	Breast	9	Yes	NO	1.5
9	Park et al. (12)	Female	69	Bilateral	Breast	6	Yes	NO	2
10	Rao and Choudhry (13)	Female	45	Bilateral	Breast	–	Yes	NO	Death
11	Baskin and Garg (14)	Female	40	Bilateral	Breast	6	Yes	NSAID and GC	4
12	Tanaka et al. (15)	Female	47	Bilateral	Breast	4	Yes	NO	2
13	Matsuoka et al. (16)	Female	39	Bilateral	Breast	8	Yes	STTA	11
14	Ehlers et al. (17)	Female	59	Bilateral	Breast	–	Yes	DRZ	1
15	Hassall and Andrew (18)	Male	73	Bilateral	Hypopharynx	2	Yes	DRZ and IVB^b^	2
16	Sridhar et al. (19)	Female	48	Bilateral	Pancreas	–	No	NO	–
17	Lee et al. (20)	Female	43	Bilateral	Pancreas	4	Yes	NO	3
18	Ota et al. (21)	Male	71	Bilateral	Pancreas	–	Yes	NO	2
19	Ito et al. (22)	Female	73	Left Eye	Pancreas	4	Yes	NO	6
20	Burgos-Blasco et al. (23)	Male	67	Bilateral	Pancreas	6	Yes	DEX	4
21	Otsubo et al. (24)	Female	72	Bilateral	Breast	2	Yes	DRZ	1.5^c^
22	Otsubo et al. (24)	Male	70	Bilateral	Pancreas	5	Yes	DRZ	1.25
23	Present case	Female	45	Bilateral	Breast	18	Yes	DRZ and IVR	2

NSAID, topical nonsteroidal anti-inflammatory drug; DEX, intravitreal dexamethasone; DRZ, topical dorzolamide; IVB, intravitreal bevacizumab; GC, topical corticosteroids; STTA, subtenon triamcinolone acetonide injection; IVR, intravitreal ranibizumab.

a IVB applied every 4 weeks: two injections for the right eye and three for the left.

b DRZ for the right eye and IVB monthly for the left eye.

c A total of 1.5 months for the right eye and 2.5 months for the left eye.

In these cases, the male to female ratio was 1:2.8, the mean age was 60 years (32–73 years), and almost all had bilateral eyes involvement except one. Fourteen cases were diagnosed with breast cancer (7–17, 24), six with pancreatic cancer (19–24), and the others with melanoma (5), cancer of the hypopharynx (18), and lung cancer (6), respectively. The treatment regimens were heterogeneous, with most patients discontinuing nab-paclitaxel with or without eye treatments, including topical nonsteroidal anti-inflammatory drugs (NSAIDs), topical corticosteroids, topical dorzolamide (DRZ), intravitreal bevacizumab (IVB), dexamethasone. Our case was the first patient treated with ranibizumab. We analyzed the recovery time course of all the cases, which are listed in **Table 1**. The recovery period in these cases was from 3 weeks to 11 months, with a mean period of 2.8 months. In the cases without other treatments besides the cessation of nab-paclitaxel, the macular edema subsided within 1.5–6 months (2.6 months on average, *n* = 10), while the cases treated with DRZ seemed to experience a shorter recovery duration of 1–2 months (1.65 months on average, *n* = 6). Moreover, the cases treated with oral glucose corticoid or intravitreal dexamethasone seemed to experience a longer resolution duration (0.75 to 11 months, *n* = 3).

## Discussion

Taxanes, including docetaxel, cabazitaxel, paclitaxel, and nab-paclitaxel, are microtubule-stabilizing agents that are clinically effective against various malignancies (25). Paclitaxel was the first member of the taxoid family and was approved for use in chemotherapy in 1993 (26). Due to its hydrophobic nature, paclitaxel is poorly soluble. Its first licensed formulation used polymethylated castor oil (Cremophor EL, CrEL) and ethanol paclitaxel emulsification. However, CrEL can form tiny particles encapsulating paclitaxel molecules, affecting the absorption and utilization of tumor tissues. In addition, the degradation of CrEL may cause hypersensitivity reactions, requiring prolonged infusion times and premedication with steroids and antihistamines (27). Nab-paclitaxel is a newer formulation that uses albumin-bound nanoparticles to enhance paclitaxel solubility (2). A phase III study suggested that nab-paclitaxel has shown better efficacy in antitumor with fewer adverse effects when compared to paclitaxel (28). Thus, albumin-bound paclitaxel is widely used in the chemotherapy of malignant tumors.

T-CME is a rare side effect of taxanes, with an incidence of about 0.2–0.5% (3, 4). The first case of T-CME was reported in 2003, caused by docetaxel (29). Then, CME caused by paclitaxel and nab-paclitaxel was first reported in 2007 (30) and 2008 (9), respectively. Nab-paclitaxel-related T-CME is mostly bilateral, with onset at 9.3 months (range: from 1.5 to 42 months) after applying albumin-bound paclitaxel.

It has been reported that, in 97.83% of the cases, no leakage was detected in fundus fluorescence angiography (FFA) or significant ICGA changes (20, 31). In all T-CME cases listed here (literature review and case report), which resulted from albumin-bound paclitaxel, no or minimal leakage was identified by FFA. As shown in the structural OCT B-scan, the hyporeflective cysts were located in the outer nuclear layer and inner nuclear layer, with intact contiguous outer plexiform layer and outer plexiform layer and retinal pigmented epithelium. Unlike CME caused by retinal vein occlusion or diabetic retinopathy, hard exudation in fundus photographs and hyperreflective plaques in OCT were not seen in T-CME cases, coinciding with the previous study (32). Taken together, increased capillary permeability may not be the primary cause of T-CME. Furthermore, though limited cases underwent OCTA examination, intact parafoveal capillary networks were detected without blood flow interruption. It indicated that T-CME might not be a result of vascular degeneration of vessel occlusion.

Another hypothesis is that T-CME may associate with fluid retention syndrome, which is frequently seen in doxorubicin therapy and involves peripheral edema, weight gain, and fluid accumulation in the third space (pericardium, pleura, and ascites) (33). However, neither the previous nor our case was mentioned with combined thoracoabdominal fluid, and the present findings are insufficient to support this hypothesis.

Concerning treatment, the literature reviewed showed that some T-CME cases spontaneously resolved after discontinuing nab-paclitaxel without eye treatment within 1.5 weeks to 6 months (5, 6, 9–13, 19–21). Though the mechanism of inflammatory factors is uncertain, anti-inflammatory agents (steroids or NSAIDs) were applied previously. Nevertheless, those drugs were reported to be ineffective, and the patients even had a longer recovery time of 4 to 11 months (4 months on average), suggesting that inflammatory factors are not the primary cause of T-CME.

Regarding the anti-VEGF treatment, only two cases were reported with IVB (8, 18) and the present case with IVR. In an interesting study conducted by Hassals et al., the patient received DRZ for the right eye and IVB monthly for the left eye, and CME subsided at around 2 months in both eyes (18). In our case, after being treated with ranibizumab, the area of the edema was narrowed. These data suggested that anti-VEGF may affect T-CME but not critically.

Interestingly, six cases (7, 17, 18, 24), including the present case, treated with a combination of topical carbonic anhydrase inhibitor (CAI), seemed to experience a shorter recovery duration (1 to 2 months). In particular, Ehlers et al. reported a monocular control trial showing that CME in the eye treated with topical CAI resolved more rapidly than the other eye without extra medication after nab-paclitaxel cessation (17). In our case, when anti-VEGF was discontinued and switched to topical CAI, the macular edema was still getting better. These cases suggested that topical CAI may also have a therapeutic effect in nab-paclitaxel-induced CME. Although we cannot confirm whether the recovery was achieved by withdrawing the drug or the efficacy of the combined treatment with ranibizumab or CAI through a retrospective case report, we could still insist that CAI may have a therapeutic effect on T-CME. Nevertheless, we should also be aware that neither anti-VEGF nor CAI plays a crucial role in treating T-CME.

The underlying pathogenesis of T-CME remains unclear. Based on a careful analysis of published literature and cases, we propose that T-CME may be attributed to the disturbed distribution of the water channels in retinal Müller cells and retinal pigment epithelium (RPE), which is caused by the inhibitory effect of paclitaxel on microtubules and ultimately leads to retinal water transport impairment. Müller cells are responsible for the osmotic gradient in the neurosensory retina surrounding the intermediate capillary plexus and deep capillary plexus vessels. These cells possess a large number of water channels (34). Since the arrangement of these channels in cells depends on microtubule function, which is altered by paclitaxel, eventual edema occurs due to impaired water transport. This hypothesis may explain the possibility of recovery with nab-paclitaxel suspension. On the other hand, carbonic anhydrase (CA) XIV, an extracellular membrane-bound CA, was identified in retinal Müller cells, astrocytes, and the apical and basolateral membranes of the RPE (35, 36). The author noted that CA XIV plays a role in pH and volume homeostasis in the extracellular space, and they suggested that CA XIV is the CAI target that enhances subretinal fluid absorption in macular edema (35). This is probably the reason why the recovery time looks shorter when combined with CAI treatment.

It is worth noting that there are also reports of no vision recovery even after macular edema has subsided after paclitaxel discontinuation (37). Moreover, in some cases of delayed CME diagnosis, macular degeneration progresses, and vision loss is irreversible (38).

## Conclusion

Nab-paclitaxel is a commonly used anti-cancer drug, which may cause macular edema. Hence, patients should undergo eye examinations routinely, including visual acuity examinations and OCT or OCTA. FFA should be applied when necessary. For oncologists, it is important to be aware of the possibility of causing CME when using nab-paclitaxel. Furthermore, attention should be paid to differentiate from CME associated with paraneoplastic syndrome. Most T-CME would recover after suspending the drug; however, long-existing CME may result in irreversible vision loss with macular degeneration, and a combination of CAI or anti-VEGF may be considered for those long-existing CME.

## Data Availability Statement

The original contributions presented in the study are included in the article/supplementary material. Further inquiries can be directed to the corresponding authors.

## Ethics Statement

Written informed consent was obtained from the individual(s) for the publication of any potentially identifiable images or data included in this article.

## Author Contributions

SY and QF drafted the manuscript. JY and JX contributed to the acquisition of the data and clinical assessment. ST and JHM analyzed the review data and critically revised the manuscript. All authors contributed to the article and approved the submitted version.

## Conflict of Interest

The authors declare that the research was conducted in the absence of any commercial or financial relationships that could be construed as a potential conflict of interest.

## Publisher’s Note

All claims expressed in this article are solely those of the authors and do not necessarily represent those of their affiliated organizations, or those of the publisher, the editors and the reviewers. Any product that may be evaluated in this article, or claim that may be made by its manufacturer, is not guaranteed or endorsed by the publisher.
